# Obesity and Overweight Prevalence Among Adolescents With Disabilities

**Published:** 2011-02-15

**Authors:** James H. Rimmer, Kiyoshi Yamaki, Brienne M. Davis, Edward Wang, Lawrence C. Vogel

**Affiliations:** Center on Health Promotion Research for Persons with Disabilities, Department of Disability and Human Development, University of Illinois at Chicago; Department of Disability and Human Development, Chicago, Illinois; Department of Disability and Human Development, Chicago, Illinois; College of Nursing, University of Illinois at Chicago, Chicago, Illinois; Shriners Hospital for Children, Chicago, Illinois

## Abstract

**Introduction:**

We examined overweight and obesity prevalence among adolescents with disabilities by disability type (physical vs cognitive) and demographic factors (sex, age, race/ethnicity).

**Methods:**

Parents (N = 662) of adolescents aged 12 to 18 years with disabilities from 49 states responded to an online survey from September 2008 through March 2009.

**Results:**

Prevalence of obesity among adolescents with physical and cognitive disabilities (17.5%) was significantly higher compared with that among adolescents without disabilities (13.0%). Obesity prevalence was higher among males, 18-year-olds, and youths with cognitive disabilities than among females, younger adolescents, and youths with physical disabilities.

**Conclusion:**

The higher prevalence of obesity among youths with disabilities compared with nondisabled youths, particularly in certain subgroups, requires further examination in future surveillance research.

## Introduction

Youths with disabilities account for 9.2%, or 6 million, school-aged children in the United States (1). The epidemic of childhood obesity observed in youths without disabilities (2) may be an even more serious health issue for youths with disabilities. Compared with youths without disabilities, youths with disabilities are generally less physically active (3) and their lifestyle health behaviors (eg, social participation, parent supervision, diet) are often markedly different (4).

Several secondary health conditions reported by youths and adults with disabilities, including chronic pain, social isolation, depression, falls or other injuries, and extreme fatigue, are likely to worsen with excess weight (5-8). Being or becoming obese in addition to having a physical or cognitive disability could impose greater demands on the person and the caregiver in performing activities of daily living and instrumental activities of daily living, and could increase health care costs associated with the multiple health effects of having a disability and being obese (9).

Previous studies in select groups of youths with disabilities (10-12) and youths who report having a chronic condition (13) have confirmed that obesity rates are higher in this population. However, there is little, if any, research that compares obesity prevalence by age group, race/ethnicity, and disability type (physical vs cognitive). The main objective of this study was to determine the prevalence of obesity and overweight among youths with disabilities by sex, race/ethnicity, age, and disability type. Results are compared with available data for youths without disabilities.

## Methods

From September 2008 through March 2009, parents who had an adolescent aged 12 to 18 years with a disability were recruited for this cross-sectional study from national and local disability and health advocacy organizations, the subscriber list of a magazine for parents of children with disabilities, and a disability-related independent website. Recruitment methods included blast e-mails, web-banner advertisements, and flyers. Eligibility criteria were having a child aged 12 to 18 years with a disability, living in the United States, and being able to speak, read, or understand English. Exclusion criteria were living outside the United States and having family members who did not read or speak English. Recruitment materials directed eligible family members (eg, parents) to visit the survey website and respond to questions regarding the health status of their child. The research protocol was reviewed and approved by the University of Illinois at Chicago institutional review board. One of the main outcomes of interest was obesity and overweight prevalence between adolescents with physical disabilities and those with cognitive disabilities.

### Instrument

Height, weight, and disability data were obtained from an online survey related to the health and lifestyles of youths with disabilities. The survey had 32 items, including height, weight, and disability classification. Only the height and weight data are reported in this study.

### Disability categories

Disability information was collected using categories of disabilities noted in the federal definition of youths who are eligible for special education services (1). Parents were asked to select up to 3 conditions associated with their child's disability from this list of conditions: autism spectrum disorder; attention deficit hyperactivity disorder; blindness or vision problems; deaf-blind; Down syndrome; epilepsy or seizures; head injury; impairment or deformity of foot or leg; impairment or deformity of hand, arm, or finger; learning disability; mental retardation (referred to in this article as intellectual disability, in light of recent initiatives to abandon the use of this term); mental or emotional problem or disorder; missing legs, feet, hands, arms, or fingers; paralysis of any kind; spina bifida; spinal cord injury; and "other."

### Body weight status

Height, weight, and birth date of adolescents, reported by a parent, were used to obtain body mass index (BMI). A percentile ranking of each adolescent's raw BMI score relative to age- and sex-specific national norms was obtained by using the criteria established by the 2000 Centers for Disease Control and Prevention Growth Charts for the United States (14). On the basis of the obtained percentile ranking, BMI status was classified into the following 4 categories: obese (BMI ≥95th percentile), overweight (BMI ≥85th percentile and <95th percentile), healthy weight (BMI <85th percentile and ≥5th percentile), and underweight (BMI <5th percentile) (15).

### Data analysis

The difference in body weight status among the study subjects by demographics and mobility status (ie, use of an aid such as a wheelchair or other assistive device) was examined by using χ^2^ tests and SPSS version 16 (SPSS Inc, Chicago, Illinois). From a descriptive framework only, we compared our data with the population-based data obtained from the 2007 Youth Risk Behavior Survey (YRBS) (15). Level of significance was set at .05.

## Results

Almost all of the family members who responded to the survey were mothers (91.2%), followed by a small number of fathers (6.5%), grandparents (1.1%), and other family or nonfamily members (1.3%). The largest reported subgroup was autism spectrum disorder, followed by cerebral palsy, Down syndrome, and intellectual disability (Table 1). Males and whites were overrepresented in the study group compared with the YRBS sample.

Youths with disabilities in our survey had a higher prevalence of obesity compared with youths in the YRBS (17.5% vs 13.0%) (Table 2). In our survey, the rate for males was significantly higher than for females (19.8% vs 13.3%, χ^2^ [1, 645] = 4.3, *P* = .04). Youths with disabilities also had a higher rate of obesity than youths in the YRBS when analyzed by sex and race/ethnicity. These differences between the 2 groups across age groups were observed at ages 15 (18.9% vs 13.8%) and 18 years (25.3% vs 12.0%). Within the group of youths with disabilities, blacks and Hispanics also had a higher prevalence of obesity than other ethnic groups but the differences were not significant. The prevalence of obesity for 18-year-old youths with disabilities (25.3%) was significantly higher than that for the younger age groups with disabilities (χ^2^ [1, 645] = 4.0, *P* = .04). Youths with cognitive disabilities had a significantly higher rate of obesity compared with youths with physical disabilities (21.1% vs 10.1%, χ^2^ [1, 645] = 11.7, *P* < .001).

When compared with youths in the YRBS, youths with disabilities had a higher prevalence of overweight among females, whites, and 17-year-olds.

## Discussion

The higher rate of obesity among adolescents with disabilities compared with that among adolescents without disabilities supports the existing literature (12,16) and extends this research by identifying substantial differences between age groups, males and females, and youths with physical versus cognitive disabilities. In particular, our findings showed that the 18-year-old group had the highest obesity rates compared with the younger age groups. This is a potential indicator that obesity may be tracking into adulthood.

The significantly higher rate of obesity among youths with cognitive disabilities compared with that among youths with physical disabilities requires further research. One reason for this difference may be related to the potential error associated with BMI in youths with physical disabilities who have some form of paralysis (eg, cerebral palsy, spina bifida). Several studies on adults with spinal cord injury have confirmed that BMI is not an accurate indicator of obesity for adults with some form of paralysis (17,18). McDonald et al (19) also reported that BMI cutoff criteria in adolescents with spinal cord injury significantly underestimate obesity in this population, and they recommended that more research be done on youths with physical disabilities to determine the most appropriate cutoffs for obesity and overweight to compensate for the loss in lean body mass associated with paralysis.

Our findings are limited by the use of a convenience sample and the overrepresentation of families who were able to complete an online survey; who were from a more educated, higher socioeconomic status; and who were predominantly white. This overrepresentation limits the generalizability of our findings to the broader population of youths with disabilities. Also, the extent to which our data are directly comparable to the population-based YRBS data is not known. Future research must identify ways to target a higher percentage of minority youths with disabilities. As with most self-reported data on height and weight in adolescents and adults, there is a tendency to underestimate overweight prevalence compared with measured data (20).

The higher prevalence of obesity in youths with disabilities underscores the need to enhance data surveillance systems (eg, YRBS) using consistent disability definitions so that interventions can be targeted to youths with the greatest need. Most of the recent attention on obesity has focused on the epidemic of childhood obesity among youths without disabilities (21); less attention has been given to youths with disabilities. Decreasing the incidence of obesity among youths with disabilities must become as important a national priority as it is for youths without disabilities. Federal and private funding agencies must ensure that future obesity-related policy and environmental initiatives recognize the needs of families who have a child with a disability and who may need certain adaptations or accommodations to access existing health promotion programs offered in their schools and communities. Engaging families, and whenever possible youths with disabilities, in developing new strategies to reduce obesity must occur on the front end rather than back end (ie, after the program is installed) to provide greater assurance that new initiatives will be accessible and appropriate for youths with disabilities.

## Figures and Tables

**Table 1:** Demographic Characteristics of Adolescents With Disabilities (N = 662), 2008-2009, and Adolescents in the Youth Risk Behavior Survey, 2007

**Demographic Variable**	Adolescents With Disabilities, n (%)	YRBS Sample, %^a^
**Sex**		
Male	426 (64.4)	49.5
Female	236 (35.6)	50.5
**Age, y**		
12	90 (13.6)	NA
13	118 (17.8)	NA
14	103 (15.6)	NA
15	94 (14.2)	29.0^b^
16	84 (12.7)	26.2^c^
17	87 (13.1)	23.4^d^
18	86 (13.0)	21.3^e^
**Race/ethnicity**		
White	540 (83.3)	60.3
Black	28 (4.3)	15.1
Hispanic	31 (4.8)	16.9
Other	49 (7.6)	7.7
**Major disability groups^f^**		
Autism spectrum disorder	161 (24.4)	NA
Cerebral palsy	117 (17.8)	NA
Down syndrome	87 (13.2)	NA
Intellectual disability^g^	86 (13.1)	NA

Abbreviations: YRBS, Youth Risk Behavior Survey; NA, not applicable.

a Weighted percentage from the 2007 YRBS (14).

b Reported percentage for students in grade 9.

c Reported percentage for students in grade 10.

d Reported percentage for students in grade 11.

e Reported percentage for students in grade 12.

f Four highest reported disability groups. Remaining reported groups were spina bifida (3.3%); mental or emotional problem or disorder (3.0%); head injury (3.0%); learning disability (2.6%); epilepsy or seizures (2.4%); attention deficit hyperactivity disorder (1.8%); blindness or vision problems (1.4%); deafness and hearing problems (0.8%); deaf-blind (0.8%); missing legs, feet, hands, arms, or fingers (0.3%); spinal cord injury (0.3%); paralysis of any kind (0.2%); impairment or deformity of hand, arm, or finger (0.2%); and "other" (11.3%).

g Intellectual disability is the new term for mental retardation, which is no longer used.

**Table 2:** Percentage of Obese^a^ and Overweight^b^ Adolescents With Disabilities (N = 662), 2008-2009, and Adolescents in the Youth Risk Behavior Survey, 2007, by Selected Characteristics

Demographic Variable	Obese		Overweight	

	Adolescents With Disabilities	YRBS^c^ (95% CI)	Adolescents With Disabilities	YRBS^c^ (95% CI)
**Total**	17.5	13.0 (11.9- 14.1)	16.0	15.8 (14.8-16.8)
**Sex**				
Male	19.8^d^	16.3 (15.1-17.5)	15.5	16.4 (15.4-17.5)
Female	13.3	9.6 (8.3-11.0)	16.8	15.1 (13.8-16.5)
**Race/ethnicity**				
White	16.7	10.8 (9.3-12.4)	16.1	14.3 (12.9-15.7)
Black	25.0	18.3 (16.2-20.7)	14.3	19.0 (17.2-20.9)
Hispanic	23.3	16.6 (14.7-18.7)	20.0	18.1 (16.1-20.3)
Other	17.0	NA	14.9	NA
**Age, y**				
12	13.5	NA	18.0	NA
13	21.9	NA	18.4	NA
14	14.9	NA	15.8	NA
15	18.9	13.8^e^ (12.5-15.2)	15.6	17.6^e^ (15.5-20.1)
16	13.4	13.2^f^ (11.5-15.0)	13.4	16.0^f^ (14.4-17.6)
17	14.0	12.7^g^ (11.3-14.4)	17.4	15.1^g^ (13.3-17.1)
18	25.3^d^	12.0^h^ (10.5-13.7)	12.0	14.0^h^ (12.5-15.6)
**Disability subgroup**				
Physical disabilities	10.1	NA	12.0	NA
Cognitive disabilities	21.1^d^	NA	17.8	NA

Abbreviations: YRBS, Youth Risk Behavior Survey; CI, confidence interval; NA, not applicable.

a Body mass index ≥95th percentile.

b Body mass index ≥85th percentile and <95th percentile.

c Source: 2007 YRBS (14).

d Significantly higher compared with other disability subgroup(s) on the same demographic variable.

e Reported percentage for students in grade 9.

f Reported percentage for students in grade 10.

g Reported percentage for students in grade 11.

h Reported percentage for students in grade 12.
